# Steering opinion dynamics via containment control

**DOI:** 10.1186/s40649-017-0048-0

**Published:** 2017-11-27

**Authors:** Pietro DeLellis, Anna DiMeglio, Franco Garofalo, Francesco Lo Iudice

**Affiliations:** Department of Electrical Engineering and Information Technology, University of Naples Federico II, via Claudio, 21, 80125 Napoli, Italy

**Keywords:** Containment control, Opinion dynamics, Complex networks, Agent-based models, Pinning selection, Extremist, Proximity

## Abstract

In this paper, we model the problem of influencing the opinions of groups of individuals as a containment control problem, as in many practical scenarios, the control goal is not full consensus among all the individual opinions, but rather their containment in a certain range, determined by a set of leaders. As in classical bounded confidence models, we consider individuals affected by the confirmation bias, thus tending to influence and to be influenced only if their opinions are sufficiently close. However, here we assume that the confidence level, modeled as a proximity threshold, is not constant and uniform across the individuals, as it depends on their opinions. Specifically, in an extremist society, the most radical agents (i.e., those with the most extreme opinions) have a higher appeal and are capable of influencing nodes with very diverse opinions. The opposite happens in a moderate society, where the more connected (i.e., influential) nodes are those with an average opinion. In three artificial societies, characterized by different levels of extremism, we test through extensive simulations the effectiveness of three alternative containment strategies, where leaders have to select the set of followers they try to directly influence. We found that, when the network size is small, a stochastic time-varying pinning strategy that does not rely on information on the network topology proves to be more effective than static strategies where this information is leveraged, while the opposite happens for large networks where the relevance of the topological information is prevalent.

## Background

Who influences whom? How do leading individuals steer the opinions of the others so to gain influence in a group, subsequently increasing their decision power? How can we describe the process of opinion formation? These are pressing open questions that a wide and interdisciplinary research effort is trying to address in the last decades [1–17]. Sociologists and psychologists investigate the cognitive implications of the social interactions on opinion formation [1], whether and how a heterogeneous group can reach agreement on an issue [2], and which are the factors determining the convincing power of a social group on a subjective opinion [17]. The economists wonder how the spread of an opinion among interacting individuals may contribute to trigger financial crises and cascades [4–6], and whether it can be employed in speculative manners [7]; with a similar goal, some politicians aim to manipulate public opinion to gain and reinforce their power [8] or seek for an optimal opinion control strategy in the campaign problem [18].

Research on opinion dynamics also benefitted from the contribution of physicists, mathematicians, and engineers [9] interested in the study of complex systems. Starting from [10], where the first model of opinion dynamics in a probabilistic framework was presented, opinion dynamics models were designed by using an analogy with the Ising model in statistical mechanics [11–13].

An alternative approach is offered by sociodynamics [14, 15], which aims at building mathematical models of a variety of different phenomena within the framework of social sciences [19, 20]. Here, the opinion is modeled as a (possibly vectorial) state variable and the goal is to understand the nonlinear dynamics leading the system’s state to consensus or to fragmentation on an issue [9]. In this perspective, a possible framework stems from the consensus problem formulated and solved in the pioneering work of DeGroot [16] and Friedkin [21], which stimulated a bulk of both theoretical and application-oriented research [22–27]. Moreover, inspired by the collective behavior of animal groups [28], as fish schools or birds flocking, leader–follower consensus protocols were developed and analyzed [22, 29, 30]. This setting, which is strictly related to pinning control in complex networks [31–36], well suits opinion dynamics [37, 38], where the aim is to investigate whether the state of the system (the collective opinion) reaches consensus at the leaders’ states. However, in the presence of multiple leaders with contrasting opinions, typically their common final goal is not anymore opinion consensus on a specific value (e.g., the opinion of a given leader), but rather the containment of the followers’ opinion in a given range (e.g., they belong to the same coalition and want to win a referendum or an election) [39, 40].

Starting from the above considerations, and combining the dynamical systems approach with complex networks theory [41–43], we explore the relationship between opinion dynamics and containment control [39, 44, 45]. In control systems theory, the latter refers to a consensus-like problem in which multiple leaders, interacting with the other agents (the followers) through an influencing network, aim to drive and maintain the followers in the convex hull spanned by their states (their opinions) [45, 46]. The convex hull then becomes the mathematical representation of the *opinion range*, in which we want to *contain* the opinions of the followers. In addition, inspired by the bounded confidence models [47–49], in which an agent does not interact with agents whose opinion is too far from his, we propose an alternative model of opinion dynamics in terms of containment control, where multiple leaders populate the network and the communication is subject to a proximity rule: at each instant, node *i* will influence node *j* only if the distance between their opinion is below a state-dependent threshold. Departing from classical bounded confidence models, we consider alternative proximity thresholds, aiming at mimicking different kinds of societies permeated by different levels of extremism. Indeed, the observation of the potential consequences of the spreading of zealotry (e.g., the diffusion of terrorism) [50–52] spurred us to investigate how societies with different levels of extremism could react to the presence of multiple leaders.

In particular, in an extremist society, the most radical agents (i.e., those with the most extreme opinions) have a higher appeal and are capable of influencing nodes with very diverse opinion. The opposite happens in a moderate society, where the more connected (i.e., influential) nodes are those with an average opinion. Finally, we call neutral a society which is neither extremist nor moderate. Therefore, to evaluate how the leaders can control each of these artificial societies, we present and numerically compare different kinds of containment strategies, that is, different ways in which the leaders try to influence the opinion of the followers. In particular, some of them leverage information on the network topology (as for instance in [53]), while others benefit from the possibility of changing the set of followers the leaders try to influence.

We find that static control strategies only marginally benefit from the knowledge of the topological properties of the network. On the other hand, we observe a relevant increase of the performance in terms of the fraction of contained followers when the leaders are allowed to rewire the network of potential connection with the followers. In other words, if a leader can change at every time instant the followers they seek to directly influence, they become able to drag more opinions in the range of interest defined by the convex hull of the their opinion.

The remainder of our work is organized as follows: first, we model the problem of steering the opinion in a group of individuals as a containment control problem, and then we introduce the alternative containment strategies to control three artificial societies with their respective proximity rules. Afterwards, we describe the numerical setup and illustrate the results. Finally, we draw the conclusions and identify future directions.

## Containment control and opinion dynamics

**Fig. 1:**
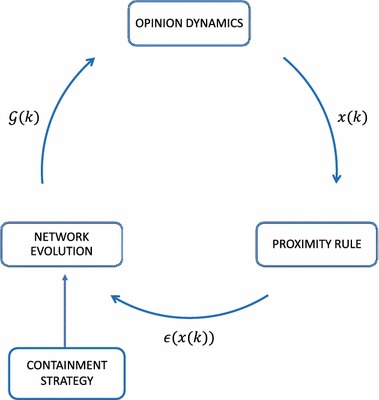
Schematic of the coevolving network describing the interplay among the opinion dynamics, the proximity threshold, $\epsilon (x(k))$, and the interaction graph, $\mathcal {G}(k)$

Here, our aim is to describe opinion dynamics in the framework of agent-based modeling. More specifically, we consider the case in which selected members of the society, that we will call *leaders*, try to steer the opinions of the other individuals, called *followers*. To this aim, we associate each individual to a node of a time-varying graph $\mathcal {G}(k)=\lbrace \mathcal {V}, \mathcal {E}(k)\rbrace$, where $\mathcal {V}$ is the set of nodes (the individuals) and $\mathcal {E}(k)$ is the edge set, representing the influence relationships at time *k*. The set of nodes $\mathcal {V}$ is partitioned in two non-empty sets $\mathcal {L}$ and $\mathcal {F}$ that are the sets of leaders and of followers, respectively. We constrain $\mathcal {G}(k)$ to be a subgraph of $\mathcal {P}=\lbrace \mathcal {V}, \mathcal {E}_{\omega }\rbrace$, which describes the potential interaction among nodes, with typically $\mathcal {E}_{\omega }\subset \mathcal {V} \times \mathcal {V}$, as the influence process is selective [54–56].

The state of a node, say *i*, represents the opinion of the *i*-th individual on a given matter of interest at time *k*, and is quantified by a scalar $x_i(k) \in \mathcal {O}:=[x_{\min },x_{\max }] \subset \mathbb {R}$, where $\mathcal {O}$ is the set of possible opinions. The state evolution of node *i* is given by

1 $$\begin{aligned} x_i(k+1)= \left\{ { \begin{array}{ll} {x_i(k)}& \quad {}{\text {if} \, i\in \mathcal {L} , }\\ {x_i(k)+ \dfrac{1}{\sigma _i(k)} \sum _{j \in \mathcal {N}_i(k)}(x_j(k)-x_i(k))}& \quad {}\text {otherwise,} \end{array}} \right. \end{aligned}$$

where $\sigma _i(k)=\max \left\{ \left| \mathcal {N}_i(k)\right| ,1\right\}$, $\mathcal {N}_i(k):=\lbrace j \in \mathcal {V} \mid a_{ji}(k)=1 \rbrace$ being the in-neighbors set of *i*, with $a_{ji}(k)$ being the *ji*th element of the time-varying binary adjacency matrix *A*(*k*) associated to the interaction graph $\mathcal {G}(k)$.

In what follows, we assume that the adjacency matrix *A*(*k*) coevolves with the node dynamics (see Fig. 1) according to a proximity rule:

2 $$\begin{aligned} a_{ji}(k)= \left\{ { \begin{array}{ll} {1}&{}{\text {if }(j,i)\in \mathcal {E}_{\omega } \quad \text { and }  \quad \mid x_j(k)-x_i(k) \mid \le \epsilon \left( x_i(k)\right) , }\\ {0}&{}\text {otherwise,} \end{array}} \right. \end{aligned}$$

where $\epsilon (x_i(k))$ is a (possibly) state-dependent threshold. Differently from classical bounded confidence models [47–49], with which models (1), (2) share the concept of *confidence level*, we have that

1. the interaction among the agents is selective [54]: two agents, say *i* and *j*, may interact only if $(i,j)\in \mathcal {E}_{\omega }$;
2. there are opinion leaders, who try to influence the opinion of the others but never adjust theirs and
3. aim at *containing* the opinion of their neighbors in the convex hull of their opinions, without requiring the convergence towards a given consensus value.

In particular, point (3) implies that we study the problem of steering the opinions of a group of individuals as a containment control problem rather than a leader–follower consensus problem. Indeed, while the diffusive protocol well describes the process of opinion spreading in a group, it is sometimes unrealistic to assume full cooperation among the leaders with the aim of steering the group opinion towards the same asymptotic values. Indeed, in presence of polarization, antagonism, speculative behaviors, and cognitive biases, ideal cooperation is unrealistic [57], and therefore we allow for the existence of multiple leaders with contrasting opinions.

To clarify how point (3), together with points (1) and (2), impacts on the time-varying network topology, we notice that the binary adjacency matrix $\bar{A}(k)$ associated to the potential graph $\mathcal {P}=\{\mathcal {V},\mathcal {E}_\omega \}$ can be decomposed in the following blocks:

$$\begin{aligned} \bar{A}(k) = \left[ { \begin{array}{ll} \mathbf {0}_{l\times l}&{}{A_{\mathcal L \rightarrow \mathcal F}(k)}\\ \mathbf {0}_{f\times l}&{}{A_{\mathcal {F} \leftrightarrow \mathcal {F}}(k)} \end{array}} \right] , \end{aligned}$$

where $A_{\mathcal L \rightarrow \mathcal F}\in \mathbb {R}^{l\times f}$ describes the possible connection (i.e., direct influence) of the leaders on the followers, while $A_{\mathcal {F} \leftrightarrow \mathcal {F}}\in \mathbb {R}^{f\times f}$ describes the possible mutual connections among the followers, with $l=\left| \mathcal L \right|$ and $f=\left| \mathcal F\right|$.

In what follows, we assume $A_{\mathcal {F} \leftrightarrow \mathcal {F}}$ to be given and constant, while we will focus on the selection of $A_{\mathcal L \rightarrow \mathcal F}(k)$. In other words, in this paper we want to compare alternative selections for $A_{\mathcal L \rightarrow \mathcal F}(k)$, given a constraint on

$$\begin{aligned} d_{\text {out}}^{\max }:=\Vert A_{\mathcal L \rightarrow \mathcal F}(k)\Vert _1 \end{aligned}$$

(i.e., on the maximum out-degree of each leader), in terms of their ability in steering the opinions of the followers in the convex hull spanned by the leaders. Also, we define the set of pinned followers at time *k* as

$$\begin{aligned} \mathcal {F}_{P}(k)= \left\{ j \in \mathcal {F}: \,\sum _{i} (A_{\mathcal L \rightarrow \mathcal F} (k))_{ij}>0\right\} . \end{aligned}$$

To formally state the problem, we first need to give some definitions.

### **Definition 1**

The convex hull $\mathbf {Co}(\mathcal {S})$ of a finite set of points $\mathcal S=\{x_1$, $\ldots , x_m\} \subset \mathbb {R}$ is the minimal convex set containing all points $z\in \mathcal {S}$, that is, $\mathbf {Co} (\mathcal {S}) =\lbrace z= \sum _{i=1}^{m} \alpha _i x_i | \alpha _i \geqslant 0, \sum _{i=1}^{m} \alpha _i =1\rbrace$.

### **Definition 2**

The opinion of the *i*th follower is asymptotically contained to those of the leaders if

$$\begin{aligned} c_i:=\left[ \liminf _{k\rightarrow +\infty } x_i(t), \limsup _{k\rightarrow +\infty } x_i(k) \right] \subseteq \mathbf {Co}(X_l), \end{aligned}$$

where $$X_l=\left\{ x_i(0)\right\} _{i\in \mathcal {L}}$$.

### **Definition 3**

Networks (1), (2) achieve q-partial opinion containment if

$$\begin{aligned} \bigcup _{i\in \mathcal {Q}}c_i\subseteq \mathbf {Co}(X_l), \end{aligned}$$

for some $\mathcal {Q}\subseteq \mathcal {F}$ such that $\left| \mathcal {Q}\right| =q$. If $q=f$ ($\mathcal {Q}=\mathcal {F})$, full containment is achieved.

Assuming that the maximum out-degree of each leader is constrained, that is, $d_{\text {out}}^{\max }(k)=\bar{d}<f$, and that the number *l* of leaders is given, we compare alternative pinning strategies to test whether they can significantly improve the fraction of contained followers, $\phi = \dfrac{q}{f}$, if compared with the trivial strategy of randomly picking a constant set $\mathcal {F}_{P}^{r}$ of pinned nodes, that is, $\mathcal {F}_{P}(k)=\mathcal {F}_{P}^{r}$ for all *k*. This comparison will be made under different assumptions on the society, that is, under different assumptions on the possibly state-dependent threshold $\epsilon$ introduced in Eq. (2).

## Containment strategies and society models

### Alternative containment strategies

Here, we present two static containment strategies, in which the pinned followers are selected at the onset of the simulation, and a time-varying containment strategy, where the set of pinned nodes is updated at every time instant.

- *St.1.* *Pinning the hubs* The set of pinned nodes is static and is composed by the set $\mathcal {F}_{P}^h$ of the $d_{\text {out}}^{\max }l$ nodes with highest degree (the hubs), that is, $\mathcal {F}_{P}(k)=\mathcal {F}_{P}^{h}$ for all *k*.
- *St.2.* *Pinning the leaves* The set of pinned nodes is static and is composed by the set $\mathcal {F}_{P}^{\ell }$ of the $d_{\text {out}}^{\max }l$ nodes with the lowest degree (the leaves), that is, $\mathcal {F}_{P}(k)=\mathcal {F}_{P}^{\ell }$ for all *k*.
- *St.3.* *Time-varying random pinning* Inspired by [32], under this time-varying containment strategy each leader, at every time instant, randomly pins a set $\mathcal {F}_{P}^{r}(k)$ of $d_{\text {out}}^{\max }l$ followers, that is, $\mathcal {F}_{P}(k)=\mathcal {F}_{P}^{r}(k)$ for all *k*.^1^

As anticipated above, the effectiveness of these containment strategies will be tested against the static random strategy

- *St.0.* *Static random pinning* The set of pinned nodes is static and is composed by the set $\mathcal {F}_{P}^r$ of the $d_{\text {out}}^{\max }l$ randomly selected nodes, that is, $\mathcal {F}_{P}(k)=\mathcal {F}_{P}^{r}$ for all *k*.

Also, as a further reference, we will consider the case in which the leaders do not try to influence any follower, that is, $\mathcal {A}_{\mathcal {L}\rightarrow \mathcal {F}}=\mathbf {0}_{l\times f}.$

We emphasize that all the containment strategies only determine the set of nodes that are potentially influenced by the opinion of the leaders, while the influence will be actually exerted only if the proximity condition is fulfilled, and this also depends on the selected society model, in agreement with Eq. (2). In other words, we assume that the leader can only decide which agents he *tries* to influence, but there is no guarantee he will be actually capable of influencing them.

### Alternative society models

The containment strategies presented above will be tested in three agent-based society models differing for the choice of the proximity threshold $\epsilon (x_i(k))$. These artificial societies are populated by agents affected by the *confirmation bias* [58], an ubiquitous psychological phenomenon that makes the agents reluctant to be influenced by individuals whose opinions are too different. Indeed, they are anchored to their beliefs and opinions and avoid the contrasts and the comparison with the diversity. However, the three scenarios are characterized by different levels of extremism, which translates into a different appeal of moderate (extremist) agents, which are agents with opinions closer to (further from) the average. Before proceeding with the description of the alternative scenarios, it is worth noticing that Eq. (1) can be rewritten as

$$\begin{aligned} x(k+1)=\tilde{A} (k)x(k), \end{aligned}$$

where $x(k)=[x_1(k),\ldots ,x_{l+f}(k)]^T$, and $\tilde{A}(k)$ is row-stochastic. Therefore, as the initial opinions are bounded in the set $[x_{\min }, x_{\max }]$, we know that the opinions of each agent will remain in that set for all *k* [59]. Therefore, we define the center $\bar{x}= \left( x_{\max }+x_{\min }\right) /2$ of this interval as the average among all the possible opinions at time 0.

#### Moderate society

In a moderate society, we assume that opinions closer to $\bar{x}$ are accounted by a larger fraction of agents if compared with more extremist opinions (i.e., closer to $x_{\min }$ or $x_{\max }$). This mechanism mimics the opinion dynamics that often characterizes the society in western democracies [60, 61]. To model this behavior, in a moderate society we set the proximity threshold that quantifies the ability of an agent to influence others, as

3 $$\begin{aligned} \epsilon \left( x_i(k)\right) = \rho \min \left( |x_{\max }-x_i(k)|,|x_{\min }-x_i(k)|\right) , \end{aligned}$$

where $\rho >0$ is a parameter determining the average radius of interaction. Notice that $0\le \epsilon \left( x_i(k)\right) \le \rho (x_{\max }-x_{\min })/2$, with $\epsilon \left( x_i(k)\right) =0$ when $x_i(k)=x_{\min }$ or $x_i(k)=x_{\max }$, while $\epsilon \left( x_i(k)\right) = \rho (x_{\max }-x_{\min })/2$ when $x_i(k)=\bar{x}$.

#### Extremist society

Historical examples [62], together with the empirical evidence [63, 64] that extreme positions are sometimes the most effective in leading the opinions in a desired range, motivated us to also model an extremist society in which the proximity threshold is

4 $$\begin{aligned} \epsilon \left( x_i(k)\right) = \rho |x_i(k)-\bar{x}|. \end{aligned}$$

This scenario is diametrically opposed to the previous one. Indeed, the maximum value $\rho (x_{\max }-x_{\min })/2$ of $\epsilon$ corresponds to extremist opinions (either $x_i(k)$ equal to $x_{\max }$ or $x_{\min }$), while a null amplitude of $\epsilon$ corresponds to $x_{i}(k)=\bar{x}$.

#### Neutral society

In an intermediate society, which we denote *neutral* and is closer to the classical bounded confidence model, the proximity threshold is independent from the agents opinion, and given by

5 $$\begin{aligned} \epsilon \left( x_i(k)\right) = \epsilon = \dfrac{\rho }{2} \left( \dfrac{x_{\max }- x_{\min }}{2} \right) . \end{aligned}$$

##### *Remark 1*

We point out that our definition of extremism differs from that given in [65–67], where it is considered as an attribute of the opinion, and therefore of the single agent. Differently, in our work the extremism is an attribute of the society, which can be measured by quantifying the appeal of extremist opinions.

##### *Remark 2*

The selection of the proximity threshold in the three scenarios is such that if the initial conditions are taken from a uniform distribution between $x_{\min }$ and $x_{\max }$, the expected value for $\epsilon \left( x_i(0)\right)$ is the same and equal to

$$\begin{aligned} \dfrac{\rho }{2} \left( \dfrac{x_{\max }- x_{\min }}{2} \right) . \end{aligned}$$

## Numerical analysis

Here, we design a set of numerical simulations to test the effectiveness of the alternative containment strategies in the three agent-based society models introduced above. We start by noticing that containment can be more or less easy to attain depending on the absolute and relative location of the leaders. Indeed, (i) the more packed their opinions are (the smaller their convex hull is), the more difficult the control goal will be, and (ii) depending on the considered scenario, also their distance from the average opinion may impact their capability of influencing (and then, of containing). Therefore, denoting by $w(\cdot )$ the width of an interval, we introduce the *leaders’ disagreement*

6 $$\begin{aligned} \delta _{\ell }= w(\mathbf Co (X_{\ell }))=\max _{i \in \mathcal {L}}x_{i}-\min _{i \in \mathcal {L}}x_i, \end{aligned}$$

which is the higher the more the opinions of the leaders are spread, and the *leaders’ polarization*

7 $$\begin{aligned} p_{\ell }= \dfrac{1}{|\mathcal {L}|} \sum _{i=1}^{|\mathcal {L}|} (x_i - \bar{x}), \end{aligned}$$

which measures the average difference between the leaders’ opinions and $\bar{x}$.

To test the effectiveness of the proposed containment strategies, we performed extensive numerical simulations for a selected combination of $\delta _{\ell }$ and $p_{\ell }$.

**Fig. 2:**
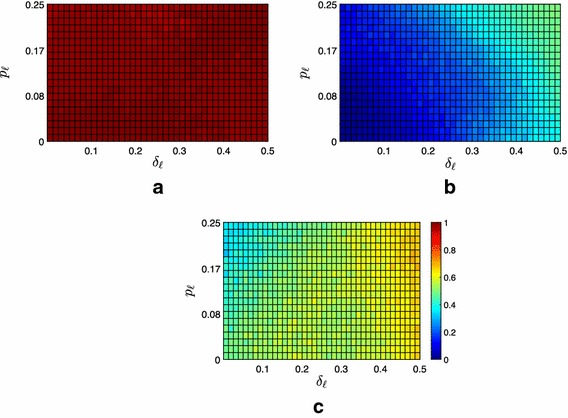
Average fraction of the $f=100$ followers contained in the convex hull spanned by the leaders under Strategy 0. **a** Moderate scenario, **b** extremist scenario, **c** neutral scenario

**Fig. 3:**
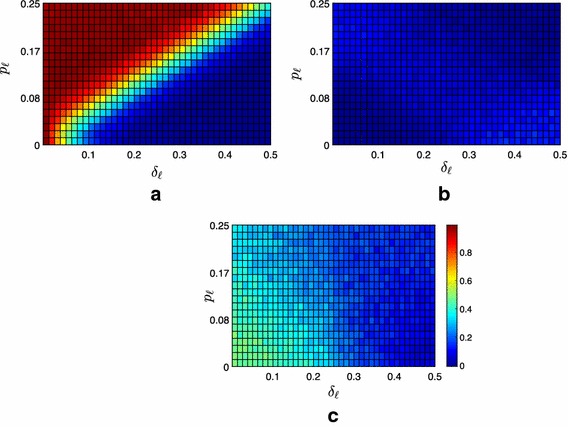
Difference between the average fraction of the $f=100$ followers contained under Strategy 0 and the case $\mathcal {A}_{\mathcal {L}\rightarrow \mathcal {F}}=\mathbf {0}_{l\times f}$ (i.e., the leaders cannot influence the followers). **a** Moderate scenario, **b** extremist scenario, **c** neutral scenario

**Fig. 4:**
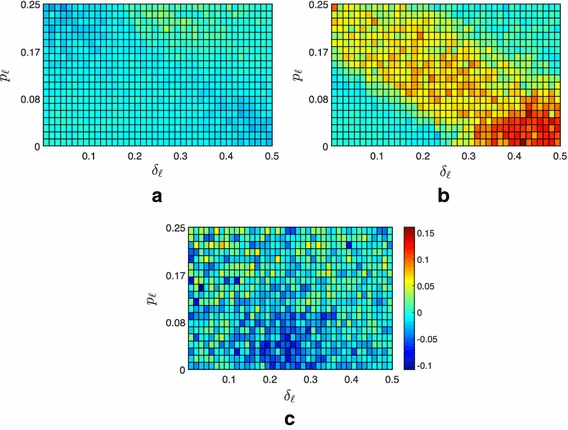
Difference between the average fraction of the $f=100$ followers contained under Strategy 1 and under Strategy 0. **a** Moderate scenario, **b** extremist scenario, **c** neutral scenario

**Fig. 5:**
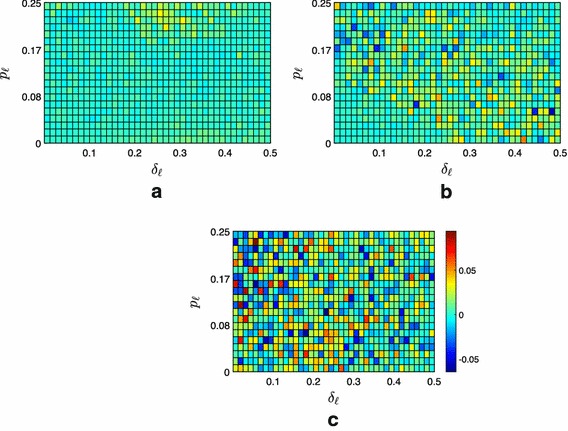
Difference between the average fraction of the $f=100$ followers contained under Strategy 2 and under Strategy 0. **a** Moderate scenario, **b** extremist scenario, **c** neutral scenario

**Fig. 6:**
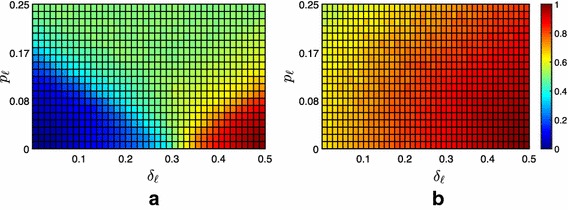
Average fraction of the $f=100$ followers contained in the convex hull spanned by the leaders under Strategy 3. **a** Extremist scenario, **b** neutral scenario

**Fig. 7:**
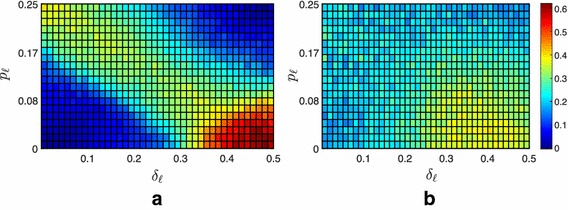
Difference between the average fraction of the $f=100$ followers contained under Strategy 3 and under Strategy 0. **a** Extremist scenario, **b** neutral scenario

**Fig. 8:**
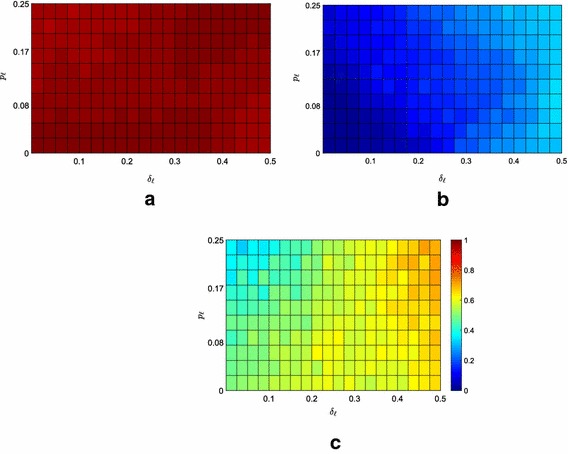
Average fraction of the $f=400$ followers contained in the convex hull spanned by the leaders under Strategy 0. **a** Moderate scenario, **b** extremist scenario, **c** neutral scenario

*Numerical setup* We consider a group populated by *f* followers with initial opinions $x_i(0)=x_{i0}$ uniformly distributed in the interval $[x_{\min }=0,x_{\max }=1]$ and $l=0.02f$ leaders. The latter are equally split in two groups with contrasting opinions, such that $\delta _{\ell }\leqslant 0.5$ and $p_{\ell }\leqslant 0.25$. Moreover, the adjacency matrix $A_{\mathcal {F} \leftrightarrow \mathcal {F}}$ corresponds to an undirected scale-free-like graph and, for each scenario, we set $\rho =1$. We consider 861 combinations for the values of $\delta _{\ell }$ and $p_{\ell }$. Namely, we vary $\delta _{\ell }$ between 0 and 0.5 and $p_{\ell }$ between 0 and 0.25, with step 0.0125, and then make the Cartesian product. Furthermore, to understand if and how the effectiveness of the containment strategies is affected by the network size, we first discuss their effectiveness when $f=100$ and then discuss what happens when the network size increases.

### $f=100$

Here, for each parameter combination, scenario, and containment strategy, we perform a set of 100 simulations differing for the randomly generated initial conditions of the followers, and evaluate the average fraction $\bar{\phi }(\delta _{\ell },p_{\ell })$ of contained followers.

#### Reference scenario: Strategy 0

Strategy 0 represents the simplest possible pinning strategy, as it is static and completely random. Indeed, it can be implemented when there is no information on the potential interconnections among the followers (i.e., on $\mathcal {A}_{\mathcal {F}\leftrightarrow \mathcal {F}}$) and when the potential influences from the leaders to the followers cannot be updated (i.e., $\mathcal {A}_{\mathcal {L}\rightarrow \mathcal {F}}(k)=\mathcal {A}_{\mathcal {L}\rightarrow \mathcal {F}}$). Therefore, we consider the performance of this strategy as a reference for the more sophisticated Strategies 1–3.

From the numerical analysis, we observe that, even though the expected width of the proximity threshold is the same for all scenarios (see Remark 2), there is a palpable difference in the fraction of followers contained that decreases together with the level of extremism in the society. From Fig. 2b, it is evident that in a society populated by moderate agents, independently of the parameters $\delta _\ell$ and $p_\ell$, in average almost all the followers are contained ($\bar{\phi }(\delta _{\ell },p_{\ell })\simeq 0.98$). Moreover, the full opinion containment ($q=\vert \mathcal {F} \vert$) is achieved in the 58% of the cases, see Table 1. Indeed, in more extremist societies, opinions tend to be clustered on the extremes, with the network graph $\mathcal {G}(k)$ often split in at least two disconnected groups, while in moderate societies opinions are easier to be contained in a given set of interest, with $\mathcal {G}(k)$ often preserving weak connectivity as *k* increases.

In scenarios other than the moderate one, the impact of the parameter $\delta _\ell$ is evident, see Fig. 2b, c. As expected, both in the neutral and in the extremist scenario, we observe that the higher is the disagreement (and therefore the width of the convex hull where we aim at containing the followers), the higher is the fraction of contained followers. This is a consequence of the fact that the control goal becomes less and less challenging as $\delta _\ell$ increases. This is clearly illustrated by Fig. 3a, which shows how the control goal for high values of $\delta _\ell$ would be achieved even if the leaders could not communicate with the followers (i.e., $\mathcal {A}_{\mathcal {L}\rightarrow \mathcal {F}}=\mathbf {0}_{l\times f}$), which is instead crucial for low values of $\delta _\ell$ and high values of $p_\ell$.

#### Strategies 1 and 2: static pinning

Here, we assume that the leaders have information on the potential relations among the followers. Specifically, we assume that the leaders are aware of potential degree of each followers, that is, they know the row-sums of $\mathcal {A}_{\mathcal {F}\leftrightarrow \mathcal {F}}$. In our numerical analysis, we tested two opposite strategies. Namely, Strategy 1 consists into pinning the followers that have the largest number of potential connections, while Strategy 2 proposes the opposite. While the rationale of the first strategy is apparent, that behind Strategy 2 is more subtle. Indeed, Strategy 2 proposes to pin the *leaves* as they are the easiest to be influenced: they will only perceive the influence of a small fraction of individuals other than the leaders.

However, the numerical analysis shows that the impact of these targeted pinning strategies is mostly negligible, see Fig. 4. For Strategy 1, we observe a noticeable effect only in the extremist scenario when $\delta _{\ell }$ is high and $p_\ell$ is low. This could be explained as a high value of $\delta _\ell$ makes the containment task relatively easier, and therefore even a small contribution from the leaders might be relevant. Moreover, the combination of a high value of $\delta _\ell$ and of a low value of the polarization $p_\ell$ makes the proximity threshold of one of the two leaders negligible, almost nullifying its contribution to the achievement of containment. In this configuration, the possibility for the more extremist leader of pinning the hubs noticeably enhances the fraction of contained followers: its influence, although mitigated by the strong connectivity of the hubs with their peers, succeeds in steering additional followers in the convex hull spanned by the leaders, see Fig. 4b.

On the other hand, analyzing the effectiveness of Strategy 2, we observe no noteworthy difference with Strategy 0, see Fig. 5. This is strictly related to the scale-free like topology of the graph (associated to $\mathcal {A}_{\mathcal {F}\leftrightarrow \mathcal {F}}$) describing the potential connections among the followers. Indeed, as the 70% of the nodes are only connected to one node (i.e., they are leaves), randomly selecting the pinned nodes (Strategy 0) is expected to be equivalent to pinning the leaves in the 70% of the simulations.

#### Strategy 3: Time-varying pinning

As observed above, the two static strategies are not capable of significantly affecting the fraction of contained followers, with the exception of Strategy 1 for specific values of the parameters in the extremist society. Therefore, we looked for alternative strategies capable of substantially improve the performance. We postulated that changing with time the set of leaders at every iteration could relevantly increase the number of followers directly affected by the leaders, thus enhancing the performance of the containment strategy. To test this hypothesis, we devised what we called Strategy 3, which prescribes to randomly select the pinned nodes at each iteration (independently from the selection at previous iterations). Notice that this simple strategy does not require any knowledge on the degree distribution of the network, differently from Strategies 1 and 2. Nonetheless, we observe a strong enhancement of the performance, see Figs. 6, 7 and Table 1, if compared to any of the static pinning strategies. Indeed, the fraction of contained followers increases for any combination of the parameters. The improvement is particularly relevant in the neutral and extremist scenarios, which are more reluctant to containment. In particular, we observe that, even in the extremist case, for favorable combination of the parameters, full containment can be achieved ($\psi >0$ in all scenarios, see Table 1). Moreover, even when full containment is not achieved, the fraction of contained followers significantly increases. For instance, in the extremist scenario, additional 20 and 23% of the total number of followers are contained if compared to Strategies 1 and 2, respectively, see again Table 1. Clearly, this strong improvement of the performance comes at the price of having to generate at every time instant the graph of the potential communication from the leaders to the followers, which means that at each time instant the leaders attempt to influence different sets of agents.

**Table 1:** Network of $f=100$ followers

Strategy	Scenario	$\bar{{\Phi }}$	$\bar{{\Psi }}$	${<d>}_{P}$
${{{\mathcal {\varvec{A}}_{\mathcal {L}\rightarrow \mathcal {F}}=\mathbf {0}_{l\times f}}}}$	Moderate	0.49	0.01	
	Extremist	0.18	0	–^a^
	Neutral	0.30	0	
St.0	Moderate	0.98	0.58	
	Extremist	0.23	0	2.00
	Neutral	0.53	0	
St.1	Moderate	0.97	0.33	
	Extremist	0.27	0	9.25
	Neutral	0.54	0	
St.2	Moderate	0.98	0.64	
	Extremist	0.24	0	1.00
	Neutral	0.54	0	
St.3	Moderate	1.00	1.00	
	Extremist	0.47	0.01	2.00
	Neutral	0.78	0.01	

For each scenario and containment strategy, we report the average fraction of contained followers ($\bar{\mathbf {\Phi }}$), the average fraction of the simulations in which full containment is achieved ($\bar{\mathbf {\Psi }}$), and the expected average degree of the pinned followers ($\mathbf {<d>}_{P}$)

^a^Here, the leaders are disconnected from the followers, and therefore there are no pinned nodes

### Increasing the network size

Here, we investigate how the network size affects the performance of the proposed pinning strategies. Indeed, when the network is small, the effectiveness of the static strategies may be limited by the fact that the difference between the lowest and highest node degrees is reduced because of the network size. This observation is confirmed by looking at Table 1 that shows how the performance of Strategies 1 and 2 is comparable with that of the simplest and random Strategy 0. The situation instead changes when the number of nodes increases. As an example, we report in Table 2 the outcome of the simulations performed on a network of $f=400$ followers: for each scenario, pinning strategy, and parameter combination, the results are averaged over 30 repetitions. We emphasize that the results are qualitatively unchanged when further increasing the network size. As it can be observed from Fig. 8, when the number of nodes increases, the impact of the parameters $\delta _{\ell }$ and $p_\ell$ are unchanged. However, the effect of the network size is indeed noticeable when comparing static versus time-varying pinning strategies. Indeed, we observed that the advantage of adopting a time-varying strategy is lost. As for lower number of nodes, the moderate scenario results to be the least challenging for the containment strategies: even the simplest Strategy 0 proves effective in containing almost all the agents, see Fig. 8. However, when the number of nodes is increased, we observe that in the extremist scenario the static strategies become significantly more efficient than Strategy 0 and, maybe more surprisingly, also of the time-varying Strategy 3. The clear differentiation from Strategy 0 is due to the fact that now the topological information leveraged by Strategies 1 and 2 indeed matters: as the network size increases, the degree distribution starts to approach to a power law, and the degree of the *hubs* and of the *leaves* starts to become significantly different. At the same time, we observe the degradation of the performance of the time-varying strategy with the increase of the network size. Indeed, continuously switching the set of nodes a leader tries to influence is rewarding only for small networks such that the blinking control signal can quickly propagate through the network. Differently, in large networks this blinking containment strategy loses its efficacy: the advantage of randomly sending inputs to all the network nodes is indeed canceled out by the slow propagation of these signals across the network.

**Table 2:** Network of $f=400$ followers

Strategy	Scenario	$\bar{\mathbf {\Phi }}$	$\bar{\mathbf {\Psi }}$	$\mathbf {<d>}_{P}$
$\mathcal {\varvec{A}}_{\mathcal {L}\rightarrow \mathcal {F}}=\mathbf {0}_{l\times f}$	Moderate	0.49	0.01	
	Extremist	0.18	0	−^a^
	Neutral	0.30	0	
St.0	Moderate	0.98	0.42	
	Extremist	0.27	0	2
	Neutral	0.68	0.1	
St.1	Moderate	0.94	0.42	
	Extremist	0.43	0	9
	Neutral	0.67	0.12	
St.2	Moderate	0.97	0.42	
	Extremist	0.46	0	5
	Neutral	0.70	0.11	
St.3	Moderate	0.98	0.49	
	Extremist	0.36	0	10.5
	Neutral	0.66	0	

For each scenario and containment strategy, we report the average fraction of contained followers ($\bar{\mathbf {\Phi }}$), the average fraction of the simulations in which full containment is achieved ($\bar{\mathbf {\Psi }}$), and the expected average degree of the pinned followers ($\mathbf {<d>}_{P}$)

^a^Here, the leaders are disconnected from the followers, and therefore there are no pinned nodes

## Conclusions

In this paper, we have formulated the problem of steering the opinion of a group of individuals as a containment control problem. Differently from the consensus setting, we relax the aim of perfect opinions’ convergence, and study the problem of containing them in the convex hull of the opinions of a set of leaders. The containment of opinion rather than their consensus is considered to model several real scenarios, such as a referendum, where those who vote *yes* (or equivalently *no*) do not share the exact same opinion, but their opinion is *contained* in a range leading to the same final decision of voting *yes* (*no*) [40].

Departing from the classical bounded confidence models, we have considered the case in which the possibility of an individual (be him a leader or a follower) of influencing the others depends on his state, that is, on his opinion. In particular, depending on the extent of the extremism permeating the society, individuals with more extreme opinions are more or less effective in promoting their views. Therefore, we have presented three alternative society models, a moderate, a neutral, and an extremist one. Assuming the presence of two leaders in the group, we numerically explored alternative selections of the pinned nodes, that is, of the nodes the leaders try to directly influence. In all the three society models, we numerically tested the effectiveness of three alternative containment strategies, for different values of the leaders’ disagreement and polarization with respect to the average opinion. Specifically, two containment strategies are static, and prescribe to pin the most (the hubs) and the least (the leaves) connected followers, respectively. The third, instead, is time-varying and assumes to randomly select the pinned nodes at every time instant. Numerical evidence strongly supports the effectiveness of this time-varying strategy over the static ones when the network size is small ($N\approx 100$), even in the extremist society where containment is harder to achieve. This excellent performance is obtained without relying on any information on the degree distribution of the network describing the potential communications among the followers: this comes at the price of requiring the ability of changing at every time instant the set of followers the leaders try to control. However, as the number of nodes increases, the relevance of the topological information becomes bigger and bigger, and therefore the informed static strategies start to outperform the non-informed time-varying strategies. Future works will (i) investigate alternative stochastic strategies to enhance the performance when local or global information on the topology of the followers is available and the number of network nodes increases; (ii) test the strategies in mixed societies, characterized by clusters of agents with different levels of extremism; (iii) reconstruct the influence path flow to quantify the extent of the direct (due to the links among leaders and followers) and indirect (due to the paths among leaders and followers) influence power of the leaders employing information theory concepts [68–70]; and (iv) validate the model through appropriate experiments designed leveraging the potential of the so-called Citizen Science [71].

## Footnotes

1 Notice that this random extraction has no memory, i.e., $\mathbf {Pr}(a_{ij}(k+1)=a\mid a_{ij}(k)=b)=\mathbf {Pr}(a_{ij}(k+1)=a)$ for all $a,b\in \left\{ 0,1\right\}$, and thus $\mathcal {F}_P(k+1)$ is independent from $\mathcal {F}_P(k)$.
